# System-Level Tensions in Scaling Digital Mental Health: A Qualitative Study of STARS in Jordan

**DOI:** 10.3390/healthcare14182986

**Published:** 2026-09-12

**Authors:** Latefa Ali Dardas, Anne Marijn de Graaff, Amjad Al-Khayat, Ammar Ali, Rayan Saleh, Rand Habashneh, Dharani Keyan, Sarah Fanatseh, Aemal Akhtar, Adnan Abualhaija, Muhannad Faroun, Ibrahim Said Aqel, Hadeel Alfar, Chiara Servili, Richard Bryant, Kenneth Carswell

**Affiliations:** 1 Deanship of Scientific Research, The University of Jordan, Amman 11942, Jordan; 2 Department of Noncommunicable Diseases and Mental Health, World Health Organization, 1211 Geneva, Switzerland; 3 Department of Educational Sciences, Salt Faculty, Al-Balqa’ Applied University, Al-Salt 19117, Jordan; 4 School of Medicine, The University of Jordan, Amman 11942, Jordan; 5 Institute for Family Health, King Hussein Foundation, Amman 11910, Jordan; 6 School of Psychology, University of New South Wales, Sydney, NSW 2052, Australia; 7 Department of Clinical Neuroscience, Division of Insurance Medicine, Karolinska Institutet, 171 77 Stockholm, Sweden; 8 Jordan Country Office, World Health Organization, Amman 11181, Jordan

**Keywords:** digital mental health, task sharing, qualitative implementation research, system innovation, youth mental health, low- and middle-income countries, Jordan

## Abstract

**Background**: Adolescents and young adults in Jordan experience high levels of psychological distress amid persistent stigma, limited mental health literacy, and shortages of specialist services. Although task-shared interventions have expanded access, their dependence on in-person delivery and sustained human resources constrains scalability. Guided digital interventions may extend reach, but evidence regarding their system integration and sustainability in low- and middle-income countries remains limited. **Purpose**: This study explored how the WHO-developed Scalable Technology for Adolescents and youth to Reduce Stress (STARS), a non-generative, rule-based chatbot intervention supported by trained e-helpers, was experienced and implemented in Jordan. It also examined the system-level tensions shaping its potential for sustainable scale-up. **Methods**: We conducted a qualitative multiple-case study nested within a randomized controlled trial of STARS in Jordan. Semi-structured interviews were conducted with 21 individuals, including STARS completers and non-completers, trained non-specialist e-helpers, a clinical supervisor, and policy and implementation stakeholders. Data were analyzed using a hybrid approach combining inductive thematic analysis with deductive coding informed by a system-innovation perspective and the culture–structure–practice framework. **Results**: Participants described STARS as culturally resonant, accessible, and adaptable to everyday life. Four recurring system-level tensions emerged: individualized support versus standardized protocols; privacy and anonymity versus institutional and parental trust; non-specialist support versus expectations for professionally qualified providers; and innovation within research settings versus integration into routine service systems. These tensions reflected competing priorities shaping implementation and perceptions of sustainable scale-up. **Conclusions**: STARS shows promise as a scalable guided digital mental health intervention for young people in Jordan. However, scale-up will require navigating recurring tensions across cultural, structural, and practice domains rather than addressing implementation barriers in isolation. This will require policy alignment, sustained investment in digital infrastructure and supervision, and cross-sector collaboration.

## 1. Introduction

Mental health conditions represent a major global public health challenge among adolescents and young people. In 2021, an estimated 279 million individuals aged 10–24 years were living with mental disorders worldwide, with depressive and anxiety disorders accounting for the largest proportion of cases. Consistent with these estimates, approximately one in seven adolescents experiences a mental health condition, and the burden is disproportionately concentrated in low- and middle-income countries (LMICs), where access to mental health services remains limited [1,2]. Jordan exemplifies this challenge. National studies have consistently documented high prevalence rates of depressive and anxiety symptoms, and distress among Jordanian adolescents and young adults, with symptom onset closely linked to academic pressure, family dynamics, social adversity, and limited economic opportunities [3,4,5,6,7]. Importantly, this body of work highlights pervasive mental health stigma, low mental health literacy, and ambivalence toward professional help-seeking, even among youth experiencing clinically significant symptoms [8]. Qualitative and mixed-methods evidence further shows that distress among Jordanian youth is frequently normalized or concealed within families, reinforcing delays in care and reliance on informal coping strategies rather than structured mental health services [5,6]. Despite this need, access to formal mental health services remains limited due to shortages of specialist providers, uneven service distribution, cost barriers, and persistent stigma surrounding mental health care [8,9]. Mental health services in Jordan are delivered through a combination of public, private, academic, and non-governmental sectors, with the Ministry of Health serving as the primary public provider. However, specialized mental health services remain concentrated in major urban centers, and dedicated child and adolescent mental health services are limited [9]. Young people typically access care through primary healthcare centers, hospitals, private clinics, or non-governmental organizations, but many do not seek formal support because of stigma, cost, lack of awareness, and concerns regarding confidentiality [8,9,10]. Consequently, a substantial proportion of adolescents and young adults experiencing psychological distress remain untreated, highlighting the need for accessible and scalable alternatives that can complement existing service structures [7,9].

Collectively, these findings underscore a substantial treatment gap and the need for youth-friendly, stigma-reducing, and scalable mental health interventions that can operate outside traditional clinic-based models in Jordan. In response, Jordan has emerged as an important implementation setting for task-shared psychological interventions delivered by trained non-specialists. A randomized controlled trial of Group Problem Management Plus (Group PM+), a WHO group psychological intervention designed for delivery by trained non-specialists [11], among Syrian refugees in a Jordanian camp demonstrated significant reductions in depressive symptoms compared with enhanced usual care [12]. In addition, a pilot study in Jordan evaluating a stepped-care program combining a guided self-help intervention (Doing What Matters in Times of Stress [13]) followed by group PM+ showed that non-specialist delivery was feasible and acceptable, with the stepped-care program being associated with greater reductions in psychological distress compared to a single brief intervention alone [14]. Task-shared interventions have also been evaluated among younger populations in Jordan. A feasibility trial of Early Adolescent Skills for Emotions (EASE) delivered by non-specialists to Syrian refugee adolescents and their caregivers demonstrated strong acceptability and procedural feasibility in humanitarian settings [15]. Subsequently, a randomized controlled trial reported significant reductions in psychological distress among adolescents receiving EASE compared with enhanced usual care at 3-month follow-up [16]. Beyond effectiveness, qualitative and process evaluations conducted in Jordan have highlighted important implementation considerations for scaling task-shared interventions. For example, evaluations of both PM+ and EASE identified factors such as community trust, perceived relevance, supervision quality, and non-specialist support as key facilitators of participation and scale-up, while stigma, competing life demands, workforce constraints, and sustainability challenges emerged as important barriers [17,18].

Complementing these trials, qualitative implementation research in Jordan has highlighted both enabling factors and persistent structural barriers to scaling task-shared psychological interventions, including workforce turnover, supervision demands, logistical constraints, and limited service reach beyond pilot settings [18]. These findings underscore a critical tension: while task-sharing has expanded access to evidence-based care, its reliance on in-person delivery and sustained human resources continues to constrain scalability within the Jordanian mental health system. Against this backdrop, digital mental health interventions have gained increasing attention as a potential strategy to extend reach, reduce stigma, and alleviate workforce pressures, particularly in low- and middle-income countries where mobile phone access is widespread and formal services remain limited [19].

Evidence suggests that guided digital interventions—those combining automated content with human support—are more effective and acceptable than fully self-guided approaches, especially for young people [20,21]. Chatbot-based interventions represent a further evolution in digital mental health delivery, offering conversational and engaging presentation of evidence-based psychological strategies while preserving standardization of content. They may also be more scalable as they can be developed on widely accessible messaging platforms, reducing the need for bespoke software design and maintenance. Emerging trials and real-world evaluations suggest that mental health chatbots are feasible, acceptable, and engaging for young people, with early evidence of symptom reduction for depression and anxiety when chatbots are grounded in established therapeutic frameworks and, in some cases, supplemented with human guidance [22,23,24]. However, most evidence on mental health chatbots derives from high-income settings, and concerns remain regarding engagement, cultural relevance, and long-term sustainability, particularly when interventions rely solely on automated interaction. For LMICs such as Jordan, the critical question is not only whether digital interventions are acceptable or effective, but whether they can be embedded within existing mental health systems, aligned with cultural norms, and supported at scale.

Within this context, the WHO–developed Scalable Technology for Adolescents and youth to Reduce Stress (STARS) intervention represents a distinct departure from prior task-shared models implemented in Jordan. Instead of delivering psychological content exclusively through face-to-face sessions, STARS delivers transdiagnostic cognitive-behavioral therapy (CBT) strategies through a non-generative, rule-based conversational agent, supplemented by human guidance from trained non-specialist “e-helpers.” Unlike PM+ or EASE, STARS shifts the primary delivery modality to a digital platform while preserving relational support through brief (15-min), structured e-helper calls. This guided self-help model builds on a growing evidence base for guided digital self-help interventions, in which structured psychological content is accompanied by brief support from trained non-specialists. Interventions such as Step-by-Step and Doing What Matters–based stepped-care programs, now detailed in the WHO manual for implementing psychological self-help interventions [25], have demonstrated effectiveness in reducing psychological distress across diverse populations, including refugees, migrants, and healthcare workers [26,27]. More recently, a published randomized clinical trial involving 344 psychologically distressed young people aged 18–21 years in Jordan demonstrated that STARS produced significant improvements across all assessed mental health outcomes, with medium-to-large effect sizes observed for anxiety and depressive symptoms [28]. Participant engagement was also high, with a mean of 7.4 (SD = 3.8) lessons completed and 66.7% of participants completing at least seven of the ten intervention lessons. These findings provide strong evidence for the effectiveness and acceptability of the STARS model in this setting and support further investigation of the implementation conditions required for sustainable scale-up and integration into routine services [28].

Existing research in Jordan has demonstrated that STARS can improve mental health outcomes and achieve substantial participant engagement under randomized trial conditions [28,29]. Research on other task-shared psychological interventions has similarly identified community trust, perceived relevance, supervision quality, and non-specialist support as important influences on participation, while also documenting workforce, logistical, and sustainability constraints [17,18]. However, this evidence does not explain how these cultural, structural, and practice-related influences interact when a guided digital intervention moves beyond a supported research setting toward routine delivery. Examining these interactions is necessary because successful scale-up depends not only on intervention effectiveness and acceptability but also on whether the intervention can be integrated, adapted, and sustained within the existing mental health system.

Accordingly, this qualitative study used a system-innovation perspective [30] and the culture–structure–practice lens articulated by van Raak [31] to explore how youth participants, e-helpers, a clinical supervisor, and policy and implementation stakeholders experienced STARS as a task-shared, digitally delivered form of mental health care in Jordan. The purpose was to identify the system conditions and recurring tensions that may shape its transition from trial-based implementation to sustainable integration within routine services. The study addressed the following research question: How do cultural, structural, and practice-related conditions interact to shape the implementation and potential sustainable scale-up of STARS within Jordan’s mental health system?

## 2. Methods

### 2.1. The STARS Intervention: Development and Delivery Model

The STARS intervention is a digitally delivered, transdiagnostic psychological intervention program developed by the World Health Organization to address common emotional distress among adolescents and young adults primarily in LMICs [32]. In the Jordanian context, STARS was adapted for young adults aged 18–21 years who reported impairing psychological distress [32]. The intervention is delivered through a rule-based conversational agent, locally named Salam, which operates using pre-programmed decision-tree logic rather than an LLM or generative artificial intelligence. This design enables standardized delivery of evidence-based CBT strategies while maintaining transparency, predictability, and clinical safety [32]. STARS is delivered across 10 structured sessions (‘lessons’) over approximately five weeks, with sessions incorporating brief mood check-ins, psychoeducation, skills practice, and reinforcement activities. Participants kept their access to the platform throughout the whole study (also after the 3 month follow-up) and that they also received 2 SMS reminders about their continued access to the platform.

STARS was developed using an extensive human-centered design process, involving iterative consultations with adolescents, young adults, community members, and mental health experts across multiple LMICs, followed by targeted cultural adaptation for Jordan [32]. This process emphasized relevance to everyday lived experiences, resulting in the use of fictional character narratives reflecting common stressors such as unemployment, family conflict, and educational pressures. Users interact with the chatbot through selectable response options, which support user agency while preserving fidelity to intervention content.

To enhance engagement and learning, sessions integrate multimodal elements, including short videos, audio exercises, quizzes, and practice activities [32]. Importantly, STARS was tested in this context with human guidance through brief, structured, weekly 15-min telephone calls with trained non-specialist e-helpers, who provide encouragement, clarify content, and support skill application without delivering psychotherapy [25]. This hybrid delivery model was designed to balance scalability with relational support, consistent with evidence that guided digital interventions are more acceptable and effective than fully self-guided formats [33].

### 2.2. Current Qualitative Study Design and Approach

We employed a qualitative multiple case study design with a systems-oriented framework analysis approach to explore the implementation and perceived impact of the STARS intervention from the perspectives of diverse stakeholders. This design was chosen for its ability to capture variation in individual and contextual experiences across multiple embedded units of analysis (e.g., participants, e-helpers, supervisors, and policy makers), while situating those experiences within the bounded case of a digital youth mental health intervention in Jordan.

This study was guided by a system innovation perspective [30] and drew on the culture–structure–practice (CSP) lens from transition theory [31] to examine system-level influences on the implementation and potential scale-up of the STARS intervention in Jordan. Within the CSP framework, culture refers to shared beliefs, values, norms, and assumptions that shape how mental health interventions are perceived; structure refers to the organizational, institutional, policy, and resource arrangements that enable or constrain implementation; and practice refers to the everyday actions, interactions, and routines through which interventions are delivered, adapted, and experienced. While Woodward et al. [30] focused analytically on culture and structure as the primary system-level conditions shaping scalability, this study extends that perspective by explicitly examining practices as the site where cultural norms and structural arrangements are enacted, negotiated, and sometimes contested. Given the qualitative and implementation-focused aims of this study, practices were therefore examined as a distinct analytic domain to capture how STARS was taken up in everyday use and how these enactments shaped perceptions of feasibility and sustainability within the Jordanian mental health context.

The CSP framework informed analysis at two complementary levels. First, it provided an initial deductive coding structure for organizing data related to cultural, structural, and practice dimensions. Second, following inductive analysis, it served as an interpretive framework through which recurring interactions across domains were examined to identify broader system-level tensions. Consequently, the four tensions reported in this study emerged inductively from participant accounts and were subsequently interpreted using the CSP framework rather than being predetermined analytical categories.

### 2.3. Sampling and Participants

A purposive sampling strategy was employed to ensure representation from key stakeholder groups central to the intervention’s delivery and reception (Table 1). The final sample included 4 STARS completers (i.e., participants who completed at least 8 chatbot lessons), 4 STARS non-completers (i.e., participants who completed less than 8 chatbot lessons), 4 e-helpers, 1 clinical supervisor who oversaw e-helper training and implementation fidelity, and 8 stakeholders (including representatives from non-governmental organizations, government institutions, and academia involved in mental health service delivery and policy).

Sampling adequacy was assessed using the principle of information power rather than numerical saturation within each stakeholder subgroup [34]. Information power was supported by the focused study aim, the participants’ direct and role-specific experience with STARS, the use of theory-informed interview guides, and the depth of the interview data. Adequacy was considered across the complete dataset and through comparison of perspectives among stakeholder groups, rather than by expecting thematic saturation within each small subgroup. The clinical supervisor was included as a key informant because of his distinct oversight of e-helper training and implementation fidelity; this role was not treated as a separate subgroup requiring independent saturation. The final sample provided both horizontal, user-level perspectives and vertical, system-level perspectives relevant to the study aim.

### 2.4. Data Collection

A total of 21 individual semi-structured in-depth interviews were conducted, with each participant interviewed once. No group or focus-group interviews were conducted. Interviews took place either in person or through a secure video-conferencing platform and lasted approximately 30–60 min each. All interviews were conducted in Arabic by trained researchers (L.AD., R.H), digitally recorded, and transcribed verbatim. Interview guides were tailored for each participant group but broadly focused on:

- Participants’ engagement with the chatbot and e-helper support,
- Perceptions of program relevance, usability, and acceptability,
- Barriers and facilitators to implementation,
- Suggestions for improvement and scaling.

The interview guides were developed collaboratively by the multidisciplinary research team, drawing on the study aims, the system-innovation perspective, the CSP framework, and prior STARS feasibility and implementation work. The team included expertise in mental health, digital intervention development, implementation research, qualitative methodology, and the Jordanian context. Team members reviewed the guides for conceptual relevance, clarity, cultural appropriateness, and consistency across participant groups, while retaining role-specific questions and prompts. During data collection, the interviewers and analysis team reviewed the initial interviews and refined the wording, ordering, and depth of selected prompts to clarify emerging issues and elicit richer accounts. These refinements did not alter the core interview domains or study aims. Changes were documented in successive versions of the interview guides to maintain an audit trail.

### 2.5. Ethical Considerations

Ethical approval was obtained from research committee of the School of Nursing, University of Jordan (PF.22.9 on 23 March 2022) and from the Ethics Review Committee at WHO (ERC.0003729 on 1 July 2022). All participants provided digital informed consent prior to participation in the qualitative interviews. The interviews adhered to protocols ensuring privacy, voluntary participation, and psychological safety. No identifiable data were retained, and pseudonyms were used in all reporting.

### 2.6. Data Analysis

Data were analyzed using a hybrid approach that combined deductive coding informed by a system innovation perspective [30] and the culture–structure–practice (CSP) lens from transition theory [31], with inductive thematic analysis used to identify emergent themes and examine enacted practices in everyday implementation. An initial coding framework was developed collaboratively by two researchers and refined after both researchers independently coded a subset of transcripts. All remaining transcripts were double-coded, and differences in coding or interpretation were resolved through discussion and refinement of the coding framework. Data-source triangulation involved systematically comparing areas of convergence and divergence across STARS completers, non-completers, e-helpers, the clinical supervisor, and policy and implementation stakeholders. Member checking was conducted with a subset of interviewees, who were invited to comment on whether the preliminary interpretations accurately reflected their experiences; their feedback was considered during the refinement of the themes. Reflexive and analytic memos were maintained throughout the analysis to document researcher assumptions, emerging interpretations, coding decisions, and changes to theme definitions. The audit trail included successive versions of the coding framework, analytic memos, records of coding discussions, and documentation of how the final themes and system-level tensions were developed. Representative quotations from different participant groups were retained to demonstrate the connection between the underlying data and the study’s interpretations. Together, these procedures strengthened the credibility, dependability, and confirmability of the analysis.

## 3. Findings

This section examines STARS as a system-level innovation situated within Jordan’s mental health ecosystem. Using a culture–structure–practice lens, we identified key systemic frictions and enabling forces that shaped successful implementation in the trial and illuminate the conditions influencing STARS’ potential for integration and scale-up.

### 3.1. Cultural Alignment, Trust, and Stigma Reduction

The first theme identified related to cultural alignment, trust and stigma reduction. To further situate these findings within a system innovation perspective, key cultural themes were analytically mapped onto the culture domain [30,31], as summarized in Table 2.

#### 3.1.1. Peer-Led Connection over Hierarchy

The presence of trained, empathetic e-helpers emerged as a central strength of the intervention. Participants consistently reported trusting e-helpers not because of professional status, but because of their relatability and perceived emotional closeness. This peer-based positioning fostered legitimacy and engagement, with weekly calls described as a primary site of support and connection. Both participants and e-helpers emphasized that these ongoing interactions were critical to motivation, emotional growth, and sustained participation.

Calls were characterized as warm, flexible, and non-judgmental. E-helpers described a sense of responsibility toward participants while maintaining professional boundaries to manage emotional demands. One e-helper reflected:

“*I felt responsible towards participants, so I was keen to encourage and motivate them to complete the sessions*.”

(E-helper 3)

Participants frequently highlighted the value of having a trusted listener and expressed a desire for longer or continued contact beyond the intervention period, underscoring the importance of relational support within the program:

“*The calls gave me plenty of space to talk about everything. The e-helpers were good listeners and responded to my questions appropriately*.”

(STARS completer 1)

#### 3.1.2. Private but Not Invisible

Participants also emphasized the importance of chatbot anonymity in reducing stigma while preserving a sense of human connection through e-helper support. The digital format was perceived as a culturally acceptable alternative for individuals who would otherwise avoid psychological services due to fear of judgment or social consequences. The chatbot provided a private space to express distress without the visibility associated with formal mental health care.

One participant noted: “*In our society, visiting the psychiatrist is not perceived well. Joining the Salam program [STARS] was one of the best choices I’ve made… I benefited a lot from talking to someone who truly understands me without judging*.”

(STARS non-completer 4)

Together, these findings illustrate how STARS offers a culturally appropriate service by combining privacy, relatability, and emotional safety, enabling participants to engage with mental health support in ways that felt socially acceptable and personally meaningful.

### 3.2. Structural Enablers and Constraints

STARS challenged structural norms in two ways: by introducing a task-sharing model via e-helpers and by delivering psychological support in a fully digital environment. These features increased access but also exposed friction points. To situate the findings within a system innovation perspective, implementation challenges were analytically mapped onto the structure domain of the culture–structure–practice (CSP) lens [30,31], as summarized in Table 3.

#### 3.2.1. Digital Accessibility Meets Digital Exclusion

One key strength of the intervention was its accessibility. This is largely attributed to low costs for participants in receiving the intervention. STARS was offered for free (as part of an RCT), and conducted online without the need to travel to face-to-face meetings.

*“Honestly, I was in need of psychological support, but I couldn’t afford to see a psychiatrist.”* STARS non-completer 3 when asked what encouraged him to participate in the program.

In addition, innovative technology used in the intervention design played a major role in attracting youth.

Participants described many factors that increased their adherence with the intervention. The chatbot’s smoothness and ease of use were consistently praised by participants, alongside having the choice to schedule the calls according to their preferences. Another factor that contributed to participants’ adherence was the simplicity of the exercises delivered through the chatbot, which were supported by a toolbox containing videos and audio files (e.g., to support practicing relaxation). Moreover, reminders were sent to participants’ mobile phones, which rendered them more engaged in the intervention.

*“Sometimes, the timing of the calls was convenient, while at other times it wasn’t. When the timing wasn’t suitable, I would let them know, and they would call me back later.”*

(STARS non-completer 4).

#### 3.2.2. Training and Supervision as Backbone

E-helpers emphasized that the five-day training program was foundational to their ability to deliver the intervention effectively. Training established a shared understanding of the intervention’s rationale, clarified roles and boundaries, and strengthened communication and teamwork skills. E-helpers described feeling confident and prepared to respond to a wide range of participant needs following training:

“*The training was exceptional. After completing it, we felt fully prepared and ready to handle any issues that might arise*.”

(E-helper 2)

While training was widely viewed as a strength, e-helpers also noted the importance of ongoing supervision to manage emotional demands and maintain intervention quality, highlighting supervision as a critical structural requirement for sustainability.

#### 3.2.3. Implementation Challenges

Despite overall positive experiences, participants and e-helpers identified several implementation challenges. Some participants required technical assistance during registration, while others found the chatbot’s response options limited or overly mechanistic. One participant noted:

“*Sometimes I feel like I am talking with a machine. I hope that the chatbot could talk more like us*.”

(STARS completer 2)

Additional challenges included grammatical errors in chatbot responses, dissatisfaction with fixed call durations, and frustration among participants who expected rapid results early in the intervention. E-helpers further described how shared phone use within households constrained communication and privacy for some participants:

“*Some participants didn’t have a personal phone and shared one with their family members*.”

(E-helper 4)

### 3.3. Practice Adaptation: From Protocol to Everyday Use

#### 3.3.1. Normalization Through Participation

Participants shared positive feedback about the STARS intervention, as they found it helpful and showed their interest in participating in similar interventions. Participants reported that the chatbot intervention helped many develop healthier coping strategies and shift their perception of mental health. Many participants said to have recommended the intervention to their friends.

“*Yes, I talked to many of my friends and neighbors about the program. I told them it was really helpful. One of our neighbors has children with disabilities, and I encouraged her to try it because she’s often anxious and stressed. I shared it with them after I finished the program and felt that I truly benefited from it, so I wanted them to benefit as well*.”

(STARS completer 4)

The STARS procedures and intervention were well received by participants, who expressed the need for psychological support that was not prioritized in their settings. Participants reported improvement in mental health, as they became more aware of themselves and more able to understand their emotions.

“*I discovered aspects about myself that I did not know before, and I am now more aware of myself*.”

(STARS completer 3).

Participants frequently mentioned that they were not only learning about new concepts but also applied them to everyday life, even when they did not complete all lessons. Whether through relaxation exercises or cognitive strategies, many found these skills useful even after the intervention ended. Beyond emotional awareness, the lessons helped participants recognize that their struggles were not unique. Knowing others faced similar challenges created a sense of shared humanity and comfort.

#### 3.3.2. Extending Practice Beyond the Platform

While STARS provided structured tools for emotional support, participants and e-helpers often extended the program beyond its original scope. Their adaptations reflect emergent, real-world practices that enhanced both personal engagement and peer diffusion—key signs of system deepening and horizontal scaling.

Several participants described sharing intervention skills—such as the relaxation exercises, identifying emotions, and reflection techniques—with family members and friends. These actions suggest early signs of behavioral diffusion, where the program’s practices extend into informal networks:

*“I even taught the exercises to my sister.”*

(STARS completer 4).

Participants recommending STARS to peers also reinforces its perceived legitimacy and personal impact, even for those who did not complete all sessions. This readiness to apply knowledge was reported even by those who disengaged before completing all lessons, reflecting the intuitive and accessible nature of the materials.

Despite the strongly protocolized e-helper support, e-helpers frequently tailored their engagement style to participants’ needs to build trust and responsiveness (e.g., adjusting their tone, interaction rhythm, and conversation depth. They suggested giving e-helpers and participants the freedom to determine the appropriate duration of the calls.

*“Some people can only talk for seven or eight minutes, while others talk for more than 20 min, and I find it difficult to ignore them and end the call, the duration of the calls should be more adjustable.”*

(E-helper 3).

Further, e-helpers expressed a need for regular supervision to manage emotional demands, especially after distressing calls. Notably, some STARS participants who discontinued the STARS chatbot lessons reported that they continued practicing exercises after disengaging, and expressed a desire for ongoing access to support tools or content. These responses highlight the intuitive, self-sustaining nature of STARS’ core practices—offering potential for asynchronous, long-term use even outside of a formal program cycle.

Recurring patterns of participant and e-helper behavior were analytically mapped onto the practice domain of the culture–structure–practice (CSP) lens, as summarized in Table 4.

In summary, STARS operated as a culturally resonant, structurally agile, and behaviorally adaptive intervention. It aligned with the lived needs of youth while skirting traditional gatekeeping systems. STARS’ impact lies not just in individual change, but in its capacity to provoke transformation across culture, structure, and practice in Jordan’s mental health ecosystem.

### 3.4. System Conditions Shaping Scalability

Stakeholders, participants, e-helpers, and supervisors described a set of interrelated conditions shaping the potential scalability and sustainability of STARS. These conditions spanned awareness and stigma, digital inclusivity, institutional coordination, funding stability, supervision capacity, and data governance. Together, they illuminate how STARS was perceived not only as an intervention, but as a system-level innovation operating within a constrained and fragmented mental health ecosystem.

#### 3.4.1. Awareness, Stigma, and Reach

Stakeholders consistently identified limited mental health awareness and persistent stigma as barriers to broader uptake, particularly among families unfamiliar with digital mental health services. Mental health was often not perceived as a priority, which constrained engagement despite expressed need. As the clinical supervisor noted:

*“One of the challenges I see is attracting the attention of young people. However, if we can raise awareness among young people that we all need this kind of support to take care of our mental well-being, I believe the program will gain more interest and engagement.”*

Participants and stakeholders described outreach strategies already used or envisioned—including social media, university-based activities, and health center displays—as mechanisms to improve visibility. Across accounts, emphasis was placed on differentiating STARS from unverified AI tools by highlighting its evidence base and the presence of trained e-helpers providing human support.

#### 3.4.2. Digital Inclusivity

While the digital format was widely viewed as a facilitator of access, stakeholders and participants also identified exclusionary barriers. Difficulties with registration, limited literacy, constrained response options, and grammatical errors in chatbot language were described as undermining credibility and engagement. Stakeholders also expressed hesitancy in implementing it, citing uncertainties around these new technologies:

“*Because it’s not a human, the participants might feel invalidated and like they’re talking to a robot. Therefore, the replies need to be humanized so that the participant doesn’t feel like they’re talking to a machine*.”

(Stakeholder 8)

These challenges highlighted a tension between stakeholders’ perceptions of how a chatbot might be received and the largely positive experiences reported by STARS participants in this study. This positive engagement was further supported by high completion and participation rates in both the chatbot-delivered lessons and e-helper calls during the RCT, suggesting strong acceptability of the digital intervention.

#### 3.4.3. Coordination, Partnerships, and System Fragmentation

Stakeholders described Jordan’s mental health system as fragmented, with limited coordination across NGOs, government entities, and academic institutions. Some stakeholders perceived that additional technical and human-resource support would be needed for the Ministry of Health to implement STARS independently and therefore emphasized the importance of coordinated partner support. At the same time, NGOs were repeatedly identified as structurally positioned to host or deliver STARS due to their proximity to communities and beneficiaries.

#### 3.4.4. Funding Instability and Sustainability

Continued funding emerged as a dominant constraint. Stakeholders described the impact of international funding cuts on organizational capacity and staffing:

*“With the USAID funding cut, our work was severely affected… we even had to reduce our staff.”*

(Stakeholder 8)

Despite this, STARS was consistently framed as potentially cost-efficient relative to traditional care, positioning it as attractive within resource-limited environments.

#### 3.4.5. Supervision, Quality, and Emotional Labor

Stakeholders emphasized that scalability was contingent on sustained supervision and support for e-helpers, particularly given emotional demands:

*“The tone of communication with e-helpers should be supportive…I suggest that any intervention should engage them with a weekly well-being activity could help alleviate their own stress, if any.”*

(Stakeholder 8)

Willingness to integrate STARS into existing services was often conditional on assurances of supervision, accountability, and quality monitoring.

#### 3.4.6. Privacy, Trust, and Data Governance

While anonymity facilitated engagement for participants, stakeholders raised concerns about data governance and child safety if anonymity was on the provider side:

“*I would never ever use programs with anonymous account administrators. I would never allow my child to use it*.”

(Stakeholder 1)

These concerns underscored the importance of transparency, consent procedures, and alignment with national regulations, something which STARS attempted to address through extensive consent procedures, clear information on who was running the intervention and accessible contact details.

### 3.5. Stakeholder-Identified Integration Pathways

Stakeholders articulated three distinct and realistic pathways through which STARS could be integrated into Jordan’s broader mental health ecosystem. These pathways reflect differing degrees of institutionalization, resource demand, and alignment with existing systems of care.

#### 3.5.1. Integration Within the Formal Mental Health System

A promising pathway involved positioning STARS as a low-intensity entry point within the formal mental health system, particularly under the Ministry of Health. Stakeholders described this model as potentially facilitating early engagement among adolescents and young adults who may otherwise avoid formal care.

*“[The] STARS program could serve as a helpful first step in facilitating access to more intensive psychological support through the Ministry of Health, particularly given the stigma surrounding mental health*.”

(Stakeholder 8)

However, this pathway was also seen as challenging given existing workforce shortages, training demands, and other constraints within the public sector.

#### 3.5.2. Integration with Humanitarian and Community Services

Many stakeholders identified NGOs, community clinics, and humanitarian programs as viable hosts for STARS. This pathway was viewed as feasible due to NGOs’ proximity to beneficiaries, existing trust relationships, and experience delivering psychosocial services in resource-constrained settings. Keeping STARS outside the formal mental health system was also perceived as potentially reducing stigma and lowering barriers to engagement.

“*NGOs represent potential entities to integrate the program, as they are closer to beneficiaries and are embedded within the community*.”

(Stakeholder 3)

At the same time, stakeholders noted that NGO capacity varies considerably, and that limited resources and resistance to adopting novel digital tools could constrain uptake in some settings.

#### 3.5.3. Community-Driven and Peer-Led Integration

A third pathway emphasized community-based diffusion through peer leaders, volunteers, or youth ambassadors. In this model, trusted individuals would promote STARS, normalize help-seeking, and facilitate engagement through informal networks and outreach activities in collaboration with NGOs. Stakeholders viewed this approach as enabling deeper social embedding of the intervention, particularly in contexts where institutional trust is limited. However, they also acknowledged that sustaining such efforts would require coordination, oversight, and ongoing support, as well as an organization or institution to host and run the software platform.

### 3.6. Synthesis of System-Level Tensions

Across stakeholder groups, the findings revealed that implementation challenges rarely occurred as isolated barriers or facilitators. Rather, participants, e-helpers, supervisors, and stakeholders consistently described competing priorities that required ongoing negotiation during implementation and would likely become more pronounced during scale-up. These recurring patterns cut across the cultural, structural, and practice domains of the CSP framework and are therefore interpreted here as system-level tensions. Rather than representing implementation failures, these tensions reflect competing demands that must be balanced to support sustainable integration of STARS within routine mental health systems.

**Figure 1:**
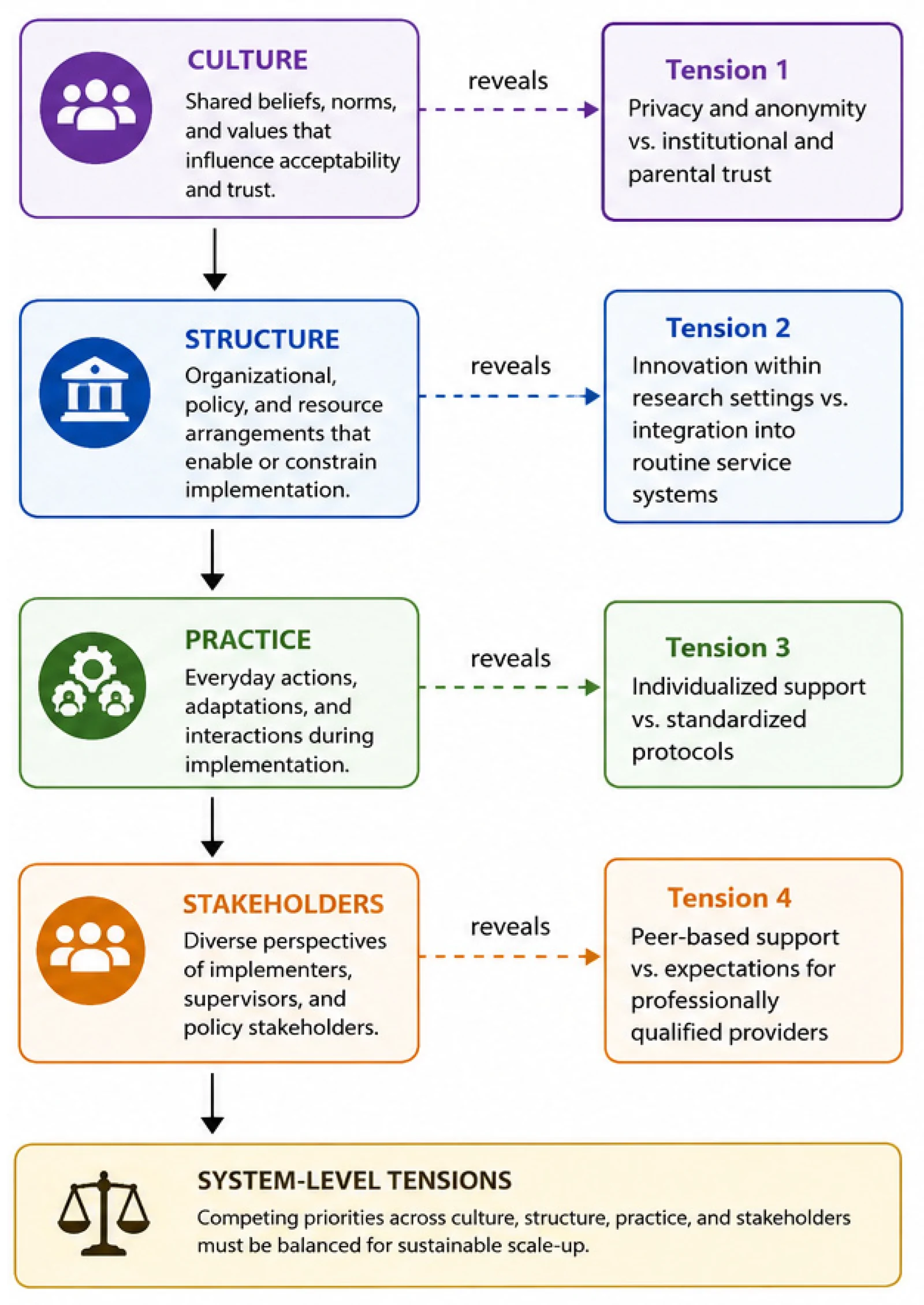
Conceptual Framework: How Domains Reveal System-Level Tensions.

The four overarching tensions are illustrated conceptually in Figure 1 and summarized, together with their practical implications, in Table 5.

As illustrated in Figure 1, the first tension—individualized support versus standardized protocols—emerged at the intersection of structure and practice. Participants and e-helpers valued flexibility in call duration, pacing, and conversational depth, whereas the intervention required standardized procedures to maintain fidelity, safety, and scalability.

The second tension shown in Figure 1—privacy and anonymity versus institutional and parental trust—linked cultural and structural considerations. Anonymity reduced stigma and made psychological support more approachable for young people; however, limited visibility regarding platform administration, data handling, and safeguarding weakened confidence among some institutional stakeholders and parents.

The third tension depicted in Figure 1—peer-based support versus expectations for professionally qualified providers—arose across the culture, structure, and practice domains. Participants valued e-helpers because they were relatable, supportive, and non-judgmental, while stakeholders emphasized the need for formal training, supervision, accreditation, and professional accountability.

The fourth tension presented in Figure 1—innovation within research settings versus integration into routine service systems—primarily reflected structural constraints. Although STARS functioned effectively with the training, supervision, monitoring, and technical support provided during the trial, routine implementation would require sustainable funding, institutional ownership, regulatory alignment, and coordination across government, academic, and non-governmental sectors.

## 4. Discussion

The principal contribution of this study is the identification of four recurring system-level tensions that shape the implementation and potential scale-up of STARS: individualized support versus standardized protocols; privacy and anonymity versus institutional and parental trust; non-specialist support versus expectations for professionally qualified providers; and innovation within research settings versus integration into routine service systems. These tensions show that cultural, structural, and practice-related influences do not function as independent barriers or facilitators. Rather, they generate competing priorities that must be negotiated as digital mental health interventions move from controlled trials into routine services. This interpretation responds to calls in global mental health to examine not only whether interventions are effective, but also how they become embedded—or fail to become embedded—within existing systems [9,35].

Because this study was nested within an RCT rather than routine service delivery, the findings should not be interpreted as evidence that STARS has already been successfully scaled, integrated, or sustained. Instead, they identify stakeholder-perceived tensions and conditions that may influence future scale-up and require prospective evaluation in real-world service settings.

### 4.1. Individualized Support Versus Standardized Protocols

Participants and e-helpers valued flexibility in the duration, pacing, and interpersonal depth of the e-helper calls, whereas STARS relied on structured content and standardized support procedures to maintain fidelity and scalability. This finding is consistent with evidence that guided digital interventions are generally more acceptable and effective than fully self-guided approaches because human support can sustain motivation and help users apply standardized content to their individual circumstances [20,21,33]. The present findings extend this literature by showing that guidance is not simply an additional intervention component; it is also the point at which standardized delivery is adapted to users’ changing needs.

More recent evidence supports this interpretation. A systematic review and meta-analysis of 29 youth chatbot studies found that chatbot-delivered interventions reduced psychological distress and were generally feasible and acceptable, although outcomes varied according to the delivery platform, interaction mode, and response-generation approach [36]. The review also identified impersonal interactions, repetitiveness, technical difficulties, privacy, and limited safeguarding as continuing concerns, reinforcing the value of combining standardized chatbot delivery with appropriately bounded human support.

E-helpers adjusted their tone, pacing, and conversational style, while participants applied STARS strategies beyond the platform, shared skills within their social networks, and continued using intervention content after disengagement. These practices indicate that meaningful uptake did not always correspond to strict completion of the intervention protocol. This supports the previous literature identifying adaptation, routinization, and everyday enactment as important mechanisms through which digital interventions become sustainable [37,38,39]. However, excessive flexibility could alter the e-helper role, increase workload, and weaken intervention fidelity. Scale-up will therefore require clearly defined components that must remain standardized, alongside bounded areas in which e-helpers may adapt the timing and style of support.

### 4.2. Privacy and Anonymity Versus Institutional and Parental Trust

The privacy afforded by the chatbot reduced the social visibility of help-seeking and allowed participants to engage with psychological support without feeling exposed to judgment. This finding is consistent with previous research showing that stigma and concerns about social consequences discourage formal help-seeking among young people and within Arab and Muslim communities [10,40]. It also supports evidence that mobile interventions may provide a more acceptable entry point for young people who are reluctant to use conventional mental health services [41]. In STARS, privacy did not produce social isolation because the chatbot was combined with regular human contact from e-helpers. Cultural acceptability therefore appeared to arise from the combination of discreet digital access and relational support rather than from anonymity alone.

The same privacy that facilitated engagement also raised concerns among stakeholders regarding platform administration, data protection, and safeguarding, particularly if STARS were extended to younger adolescents. This trust gap could be addressed through a layered consent and data-governance model. Participants could interact with the platform and e-helpers through pseudonymous identifiers, while a separately stored and encrypted identity key and audit trail could be retained for safeguarding. Access should be restricted to authorized personnel and activated only under predefined risk conditions. Consent procedures should distinguish clearly between participation, human support, data use, and emergency escalation. For implementation involving minors, age-appropriate assent and legally required parental consent should be combined with protected opportunities for confidential engagement. Transparent governance, role-based access, defined data-retention periods, and independent oversight may therefore strengthen parental and institutional trust without removing the privacy that makes the intervention acceptable to young people.

### 4.3. Non-Specialist Support Versus Expectations for Professionally Qualified Providers

Participants valued e-helpers because they were perceived as relatable, supportive, and non-judgmental rather than as distant professional authorities. This finding is consistent with task-sharing research showing that trained non-specialists can deliver acceptable and effective psychological support when their roles are clearly defined and adequately supported [35]. In the present study, e-helper contact strengthened motivation and engagement while allowing the chatbot to remain the primary vehicle for delivering psychological content. Relational support and standardized digital delivery therefore operated as complementary rather than competing intervention components.

Nevertheless, stakeholders expressed concerns about the legitimacy, accountability, and competence of non-specialist providers. These concerns show that interpersonal trust among participants does not automatically translate into institutional confidence. Training, supervision, referral pathways, and clear boundaries around the e-helper role are consequently essential for maintaining safety and quality. Participants and e-helpers in the present study described training and supervision more positively than participants in the earlier STARS feasibility trial, where adherence to e-helper calls was lower and stronger guidance, training, and supervision were recommended [29]. Refinements introduced between the feasibility and definitive trials may have strengthened e-helper confidence and participant engagement, illustrating the value of iterative implementation learning. This interpretation also aligns with wider task-sharing evidence showing that non-specialist delivery requires sustained supervision and institutional support to maintain quality and prevent emotional exhaustion [35,42]. Formal training standards, competency assessment, continuing supervision, and accreditation or mentorship pathways may help reconcile the relational advantages of non-specialist support with institutional expectations for professional accountability.

Routine implementation would require formal operating procedures that define the responsibilities and limits of e-helpers, clinical supervisors, host organizations, and technology providers. Safeguarding protocols should specify how risk is identified, documented, and escalated, including procedures for urgent consultation and referral to primary care, specialist mental health, or emergency services. Referral pathways should be mapped and tested before implementation, with clear responsibility for confirming that individuals requiring additional care have been connected to appropriate services. E-helpers should receive competency-based training and have access to a named clinical supervisor through scheduled case reviews and rapid consultation when concerns arise. Supervision should also include fidelity monitoring, workload review, and support for e-helper well-being. At the organizational level, governance procedures should address data minimization, encryption, role-based access, retention and deletion schedules, data-sharing responsibilities, and responses to privacy breaches. These structures would require alignment with national legal and regulatory requirements and evaluation under routine service conditions. These considerations align with WHO guidance on integrating evidence-based psychological interventions into existing services, including implementation planning, contextual adaptation, workforce preparation, beneficiary assessment and support, and ongoing service monitoring and evaluation [43].

### 4.4. Innovation Within Research Settings Versus Integration into Routine Service Systems

STARS lowered barriers to support through its no-cost, mobile-based format and task-sharing model. However, these advantages were demonstrated within an RCT that provided structured recruitment, training, supervision, monitoring, and technical support. Routine implementation may not reproduce these conditions. Limited internet connectivity, shared devices, limited digital literacy, unstable funding, and fragmented coordination across government, academic, and non-governmental organizations could reduce both reach and implementation quality. These findings are consistent with broader concerns that digital innovation alone cannot overcome structural inequalities within LMIC health systems [44].

Recent reviews further indicate that digital availability does not necessarily produce equitable or sustainable access. A scoping review of digital mental health interventions for adolescents in LMICs highlighted the growing potential of these interventions while emphasizing the need for context-sensitive implementation and a stronger evidence base [45]. Similarly, a systematic review found only moderate and limited evidence that digital interventions can bridge the treatment gap for socioeconomically and digitally marginalized youth [46]. Recent implementation experience also emphasizes that sustained delivery depends on organizational leadership, stakeholder involvement, workflow integration, staff training, human-support capacity, and continuous monitoring rather than technology alone [47]. Together, this suggests that digital interventions can be considered as part of a comprehensive mental health system, but should not be considered suitable for everyone, nor a replacement for in-person services.

Youth receptivity to STARS as a purpose-designed mental health intervention should also be distinguished from the effects of digital exposure more generally. Among high-school students in Kashmir, specific forms of technology use, including smartphone use, messaging, and video gaming, were negatively associated with psychological well-being [48]. These findings reflect broader concerns regarding the digital determinants of youth mental health. However, a recent WHO policy brief emphasizes that the evidence is mixed, with digital technologies showing both positive and negative associations with young people’s well-being, and that these relationships are often bidirectional and shaped by individual and environmental factors [49]. Digital reach, therefore, should not be considered inherently beneficial or harmful. Rather, its effects depend on the purpose, content, intensity, context, and safeguards of digital engagement. For purpose-designed mental health interventions, this further underscores the importance of appropriate governance, safeguarding, and integration within the broader mental health system.

Several practical strategies could support implementation under low-resource conditions. Core lessons, audio materials, and exercises could be cached for offline use and synchronized when connectivity becomes available, though the feasibility of these may in part depend on the chatbot platform being used. Partnerships with telecommunications providers could offer zero-rated access to STARS or subsidized data packages. Universities, primary healthcare centers, NGOs, and youth centers could also provide assisted onboarding and private community Wi-Fi access for users with limited connectivity, digital literacy, or personal device access. Because shared devices and community access points may introduce confidentiality risks, implementation should include discreet notifications, secure log-in and log-out procedures, private spaces, and minimal storage of identifiable information. These approaches require evaluation of their feasibility, cost, acceptability, and privacy implications before wider adoption.

The integration pathways identified by stakeholders further indicate that no single organization is likely to possess all the resources required for scale-up. Government involvement may support regulatory and overall legitimacy and connection to formal care, whereas NGOs and community organizations may offer greater proximity to young people and stronger local trust. Academic institutions could contribute training, evaluation, and implementation support. Participants’ and stakeholders’ accounts suggest that future scale-up may depend on shared ownership, stable financing, supervision capacity, and coordination across sectors; these conditions require evaluation during implementation in routine services. Collectively, the four tensions demonstrate that scale-up is not a linear transfer of an effective intervention from research to practice. It is an ongoing process of balancing flexibility with fidelity, privacy with safeguarding, relatability with professional accountability, and innovation with institutional readiness.

### 4.5. Limitations

Several limitations should be considered when interpreting the findings of this study. First, the study was nested within an RCT, which may have shaped participants’ experiences and responses. Engagement levels, perceptions of support, and implementation conditions were influenced by the presence of a research infrastructure, including structured training, supervision, and monitoring. These conditions may differ under routine service delivery, potentially affecting scalability and sustainability outside a research setting. Second, interviews relied on self-reported experiences and retrospective accounts, which are subject to recall bias and social desirability bias. Participants and e-helpers may have emphasized positive aspects of the intervention or minimized challenges, particularly given their awareness of the study’s objectives and their relationships with the research team. Third, although the study explicitly examined system-level dynamics, it did not include perspectives from all potentially relevant actors, such as parents or caregivers of participants, frontline Ministry of Health providers, or technology developers. Finally, the qualitative data capture a snapshot in time during early-stage implementation. Longitudinal qualitative follow-up would be needed to assess how perceptions, practices, and system-level tensions evolve as the intervention matures, adapts, or is integrated into routine services.

## 5. Conclusions

This study demonstrates that STARS operates not merely as a digital mental health intervention, but as a system-level innovation whose viability is shaped by the interaction of cultural norms, structural conditions, and everyday practices. While the intervention showed strong cultural resonance and practical adaptability among youth and implementers, its prospects for sustainability extend well beyond technological performance or short-term feasibility. The findings illustrate that scale-up in LMIC contexts is contingent on how digital interventions are embedded within existing governance arrangements, supervision structures, funding ecosystems, and trust relationships. Through a system innovation lens, this study advances understanding of how task-shifted, digitally delivered mental health care is negotiated within constrained and fragmented health systems. The identified system-level tensions—between personalization and standardization, anonymity and legitimacy, relational support and professional authority, and innovation and institutional integration—highlight that scalability is not a linear process but an ongoing balancing act. These tensions are not unique to STARS, but reflect broader challenges facing digital mental health initiatives in similar contexts.

Overall, STARS was perceived as acceptable and contextually adaptable under supported trial conditions. Whether these qualities can be maintained during wider implementation remains to be established through prospective evaluation in routine service settings. More importantly, the study underscores the need to conceptualize digital mental health interventions as embedded system innovations rather than standalone tools. Future efforts to scale such interventions will benefit from sustained attention to the cultural, structural, and practice-based conditions that enable—or constrain—their integration into routine care.

## Figures and Tables

**Table 1:** Interviewees’ Characteristics.

	Interviewee	Gender	Age Group
1	E-Helper 1	Female	20–29
2	E-Helper 2	Female	20–29
3	E-Helper 3	Female	30–39
4	E-Helper 4	Female	30–39
5	Clinical supervisor	Male	30–39
6	STARS completer 1	female	18–21
7	STARS completer 2	Female	18–21
8	STARS completer 3	Male	18–21
9	STARS completer 4	Female	18–21
10	STARS non-completer 1	Male	18–21
11	STARS non-completer 2	Male	18–21
12	STARS non-completer 3	Female	18–21
13	STARS non-completer 4	Female	18–21
14	Stakeholder 1	Female	60–69
15	Stakeholder 2	Female	40–49
16	Stakeholder 3	Female	30–39
17	Stakeholder 4	Male	40–49
18	Stakeholder 5	Male	60–69
19	Stakeholder 6	Male	30–39
20	Stakeholder 7	Male	50–59
21	Stakeholder 8	Female	20–29

**Table 2:** Analytic mapping of cultural findings to the culture domain of the culture–structure–practice (CSP) lens.

Subtheme	Data Element	Interpretation within the CSP Culture Domain
Peer-led connection over hierarchy	Peer-led connection over hierarchy	Shows adaptation to local cultural values (e.g., relational over clinical authority).
	Warmth of e-helper calls	Highlights valued interpersonal norms such as empathy, listening, and informal support.
Private but not invisible	Chatbot anonymity reduced stigma	Demonstrates how culturally safe formats can shift norms around help-seeking.
	Stigma, trust, and relatability	Offers lived evidence of changing beliefs and emerging cultural resonance with the intervention.
	Psychological suffering is prevalent but rarely named	Reflects prevailing mental models that avoid or minimize emotional distress.

**Table 3:** Analytic mapping of implementation challenges to the structural domain of the culture–structure–practice (CSP) lens.

Subtheme	Data Element	Interpretation Within the CSP Structure Domain
Digital accessibility meets digital exclusion	STARS challenged structural norms through task-sharing and digital delivery	Reflects innovation confronting existing structural arrangements, particularly professional hierarchies and analogue service delivery systems.
	The chatbot’s smoothness and ease of use, flexible scheduling, videos, and audio resources	Illustrates program design as a structural enabler that reduced barriers to access and improved adherence.
	Participants faced registration difficulties and required technical assistance	Indicates infrastructural weaknesses in onboarding and digital literacy.
	Fixed call duration, internet limitations, and shared phones	Highlights the mismatch between standardized protocols and real-world infrastructure.
	Chatbot limitations (grammar, restricted responses, expectation of rapid results)	Demonstrates technical and behavioural bottlenecks embedded within the intervention design.
Training and supervision as backbone	E-helpers received manualized training	Represents intentional capacity-building infrastructure supporting task-sharing.
	Training strengthened communication and teamwork skills	Reflects institutional investment in human resources for quality implementation.
	E-helpers expressed the need for ongoing supervision	Reveals gaps in structural support systems that may threaten sustainability and fidelity.

**Table 4:** Analytic mapping of cultural findings to the practice domain of the culture–structure–practice (CSP) lens.

Subtheme	Data Element	Interpretation Within the CSP Domain
Normalization through participation	Participant engagement extended beyond formal session completion	Indicates flexible uptake and non-linear engagement, suggesting that meaningful practice change does not require full protocol adherence.
	Application of intervention skills in daily life	Demonstrates enactment of intervention content in everyday practices, reflecting practice-level integration.
	Recognition of shared struggles among participants	Reflects normalization of mental health practices through collective meaning-making.
Extending practice beyond the platform	Sharing intervention strategies with family and peers	Reflects informal diffusion of practices through social networks, indicating early horizontal scaling.
	Continued use of exercises after chatbot disengagement	Suggests self-sustaining practices that persist beyond structured delivery.
	E-helpers adapting call duration, tone, and pacing	Illustrates adaptive routinization, where implementers modify formal protocols in response to user needs.
	Desire for ongoing access to intervention content	Indicates perceived value and demand for continued practice support beyond program timelines.

**Table 5:** System-level tensions influencing the scalability and integration of the STARS intervention.

Table	Description	Actionable Strategies
Care vs. Control	Youth valued emotional depth and flexibility in sessions; the program relied on standardized protocols and fixed call durations.	Further develop adaptive structures that allow e-helpers to tailor interactions while maintaining fidelity.
Visibility vs. Privacy	Anonymity helped reduce stigma and increase engagement, but it also limited institutional adoption and parental trust.	Ensure clarity around implemented layered consent and data governance, including user-facing pseudonymity, encrypted and separately stored identity records, role-based access, predefined safeguarding triggers, transparent confidentiality limits, and age-appropriate consent procedures.
Help vs. Hierarchy	E-helpers were trusted due to relatability, but lack of credentials raised concerns about quality and legitimacy.	Create accreditation or mentorship pathways that preserve empathy while meeting institutional expectations.
Innovation vs. Integration	STARS thrived in a digital, low-cost niche but lacked anchoring in existing mental health systems.	Prioritize coalition-building across NGOs, Ministry of Health, and academic institutions for sustainable integration.

## Data Availability

The qualitative data generated and analyzed during the current study are not publicly available because they contain potentially identifiable and sensitive participant information and are subject to ethical and privacy restrictions. De-identified data may be available from the corresponding author upon reasonable request and subject to approval by the relevant ethics committees and applicable data-sharing agreements.

## References

[1] Liu W., Zhang Y., Chen J., Li X., Huang Y., Zhao F., Chen F., Qu P., Li Y. (2025). Global burden and trends of major mental disorders in individuals under 24 years of age from 1990 to 2021, with projections to 2050: Insights from the Global Burden of Disease Study 2021. Front. Public Health.

[2] World Health Organization (2025). World Mental Health Today: Latest Data.

[3] Dardas L.A., Silva S.G., Smoski M.J., Noonan D., Simmons L.A. (2018). Adolescent Depression in Jordan: Symptoms Profile, Gender Differences, and the Role of Social Context. J. Psychosoc. Nurs. Ment. Health Serv..

[4] Dardas L.A., Silva S.G., Smoski M.J., Noonan D., Simmons L.A. (2018). The prevalence of depressive symptoms among Arab adolescents: Findings from Jordan. Public Health Nurs..

[5] Dardas L.A., Shoqirat N., Abu-Hassan H., Shanti B.F., Al-Khayat A., Allen D.H., Simmons L.A. (2019). Depression in Arab Adolescents: A Qualitative Study. J. Psychosoc. Nurs. Ment. Health Serv..

[6] Dardas L.A., Price M.M., Arscott J., Shahrour G., Convoy S. (2021). Voicing Jordanian Adolescents’ Suicide. Nurs. Res..

[7] United Nations High Commissioner for Refugees (2023). Annual Report 2023: Mental Health and Psychosocial Support.

[8] Dardas L.A., Simmons L.A. (2015). The stigma of mental illness in Arab families: A concept analysis. J. Psychiatr. Ment. Health Nurs..

[9] World Health Organization (2021). Mental Health Atlas 2020.

[10] Gearing R.E., MacKenzie M.J., Ibrahim R.W., Brewer K.B., Batayneh J.S., Schwalbe C.S. (2015). Stigma and mental health treatment of adolescents with depression in jordan. Community Ment. Health J..

[11] World Health Organization (2020). Group Problem Management Plus (Group PM+): Group Psychological Help for Adults Impaired by Distress in Communities Exposed to Adversity (Generic Field-Trial Version 1.0).

[12] Bryant R.A., Bawaneh A., Awwad M., Al-Hayek H., Giardinelli L., Whitney C., Jordans M.J.D., Cuijpers P., Sijbrandij M., Ventevogel P. (2022). Effectiveness of a brief group behavioral intervention for common mental disorders in Syrian refugees in Jordan: A randomized controlled trial. PLoS Med..

[13] World Health Organization (2020). Doing What Matters in Times of Stress: An Illustrated Guide.

[14] Keyan D., Habashneh R., El-Dardery H., Faroun M., Al-Johary F., Abualhaija A., Aqel I.S., Akhtar A., Dardas L., Bryant R.A. (2025). A stepped-care programme of brief psychological interventions for adults affected by adversity in Jordan: Lessons from a pilot randomised controlled trial in Jordan. medRxiv.

[15] Akhtar A., Malik A., Ghatasheh M., Aqel I.S., Habashneh R., Dawson K.S., Watts S., Jordans M.J.D., Brown F., Sijbrandij M. (2021). Feasibility trial of a brief scalable psychological intervention for Syrian refugee adolescents in Jordan. Eur. J. Psychotraumatol..

[16] Bryant R.A., Malik A., Aqel I.S., Ghatasheh M., Habashneh R., Dawson K.S., Watts S., Jordans M.J.D., Brown F.L., Sijbrandij M. (2022). Effectiveness of a brief group behavioural intervention on psychological distress in young adolescent Syrian refugees: A randomised controlled trial. PLoS Med..

[17] Woodward A., Sondorp E., Barry A.S., Dieleman M.A., Jordans M.J.D., Sijbrandij M., Cuijpers P., Roberts B. (2023). Scaling up task-sharing psychological interventions for refugees in Jordan: A qualitative study on the potential barriers and facilitators. Health Policy Plan..

[18] Terp A.M., Habashneh R., Brown F.L., Abualhaija A., Aqel I.S., Ghatasheh M., Bryant R., Jordans M.J.D., Malik A., Mittendorfer-Rutz E. (2025). Facilitators and barriers to participation and scale-up of a non-specialist delivered psychological intervention for adolescents in low-resourced settings: A process evaluation. BMC Public Health.

[19] Naslund J.A., Aschbrenner K.A., Araya R., Marsch L.A., Unützer J., Patel V., Bartels S.J. (2017). Digital technology for treating and preventing mental disorders in low-income and middle-income countries: A narrative review of the literature. Lancet Psychiatry.

[20] Baumeister H., Reichler L., Munzinger M., Lin J. (2014). The impact of guidance on Internet-based mental health interventions—A systematic review. Internet Interv..

[21] Richards D., Richardson T. (2012). Computer-based psychological treatments for depression: A systematic review and meta-analysis. Clin. Psychol. Rev..

[22] Fitzpatrick K.K., Darcy A., Vierhile M. (2017). Delivering Cognitive Behavior Therapy to Young Adults With Symptoms of Depression and Anxiety Using a Fully Automated Conversational Agent (Woebot): A Randomized Controlled Trial. JMIR Ment. Health.

[23] Inkster B., Sarda S., Subramanian V. (2018). An Empathy-Driven, Conversational Artificial Intelligence Agent (Wysa) for Digital Mental Well-Being: Real-World Data Evaluation Mixed-Methods Study. JMIR mHealth uHealth.

[24] Fulmer R., Joerin A., Gentile B., Lakerink L., Rauws M. (2018). Using Psychological Artificial Intelligence (Tess) to Relieve Symptoms of Depression and Anxiety: Randomized Controlled Trial. JMIR Ment. Health.

[25] World Health Organization (2026). Psychological Self-Help Interventions: Delivering Self-Help for Individuals, Featuring Step-by-Step and Doing What Matters in Times of Stress. https://www.who.int/publications/i/item/9789240120785.

[26] Cuijpers P., Heim E., Abi Ramia J., Burchert S., Carswell K., Cornelisz I., Knaevelsrud C., Noun P., van Klaveren C., van’t Hof E. (2022). Effects of a WHO-guided digital health intervention for depression in Syrian refugees in Lebanon: A randomized controlled trial. PLoS Med..

[27] Purgato M., Tedeschi F., Turrini G., Cadorin C., Compri B., Muriago G., Ostuzzi G., Pinucci I., Prina E., Serra R. (2025). Effectiveness of a stepped-care programme of WHO psychological interventions in a population of migrants: Results from the RESPOND randomized controlled trial. World Psychiatry.

[28] Bryant R.A., de Graaff A.M., Habashneh R., Fanatseh S., Keyan D., Akhtar A., Abualhaija A., Faroun M., Said Aqel I., Dardas L. (2026). A guided chatbot-based psychological intervention for psychologically distressed older adolescents and young adults: A randomised clinical trial in Jordan. npj Digit. Med..

[29] de Graaff A.M., Habashneh R., Fanatseh S., Keyan D., Akhtar A., Abualhaija A., Faroun M., Aqel I.S., Dardas L., Servili C. (2025). Evaluation of a Guided Chatbot Intervention for Young People in Jordan: Feasibility Randomized Controlled Trial. JMIR Ment. Health.

[30] Woodward A., Dieleman M.A., Sondorp E., Roberts B., Fuhr D.C., Ventevogel P., Sijbrandij M., Broerse J.E.W. (2021). A System Innovation Perspective on the Potential for Scaling Up New Psychological Interventions for Refugees. Intervention.

[31] van Raak R., Broerse J.E.W., Bunders J.F.G. (2010). The transition (management) perspective on long-term change in healthcare. Transitions in Health Systems: Dealing with Persistent Problems.

[32] Keyan D., Hall J., Jordan S., Watts S., Au T., Dawson K.S., Sway R.A., Crawford J., Sorsdahl K., Luitel N.P. (2025). The development of a World Health Organization transdiagnostic chatbot intervention for distressed adolescents and young adults. Front. Digit. Health.

[33] Garrido S., Millington C., Cheers D., Boydell K., Schubert E., Meade T., Nguyen Q.V. (2019). What works and what doesn’t work? A systematic review of digital mental health interventions for depression and anxiety in young people. Front. Psychiatry.

[34] Malterud K., Siersma V.D., Guassora A.D. (2016). Sample size in qualitative interview studies: Guided by information power. Qual. Health Res..

[35] Singla D.R., Kohrt B.A., Murray L.K., Anand A., Chorpita B.F., Patel V. (2017). Psychological Treatments for the World: Lessons from Low- and Middle-Income Countries. Annu. Rev. Clin. Psychol..

[36] Li J., Li Y., Hu Y., Chak Fai Ma D., Mei X., Chan E.A., Yorke J. (2025). Chatbot-delivered interventions for improving mental health among young people: A systematic review and meta-analysis. Worldviews Evid.-Based Nurs..

[37] Barak A., Hen L., Boniel-Nissim M., Shapira N. (2008). A Comprehensive Review and a Meta-Analysis of the Effectiveness of Internet-Based Psychotherapeutic Interventions. J. Technol. Hum. Serv..

[38] Andersson G., Titov N. (2014). Advantages and limitations of Internet-based interventions for common mental disorders. World Psychiatry Off. J. World Psychiatr. Assoc. (WPA).

[39] Alami H., Fortin J.P., Gagnon M.P., Lamothe L., Ghandour E.K., Ag Ahmed M.A., Roy D. (2020). Cadre stratégique pour soutenir l’évaluation des projets complexes et innovants en santé numérique [Strategic framework to Support the evaluation of complex and innovative digital health projects]. Sante Publique.

[40] Ciftci A., Jones N., Corrigan P.W. (2013). Mental health stigma in the Muslim community. J. Muslim Ment. Health.

[41] Conley C.S., Raposa E.B., Bartolotta K., Broner S.E., Hareli M., Forbes N., Christensen K.M., Assink M. (2022). The Impact of Mobile Technology-Delivered Interventions on Youth Well-being: Systematic Review and 3-Level Meta-analysis. JMIR Ment. Health.

[42] Hodgins G., Judd F., Davis J., Fahey A. (2007). An integrated approach to general practice mental health training: The importance of context. Australas. Psychiatry.

[43] World Health Organization (2024). Psychological Interventions Implementation Manual: Integrating Evidence-Based Psychological Interventions into Existing Services. https://www.who.int/publications/i/item/9789240087149.

[44] World Health Organization (2021). Global Strategy on Digital Health 2020–2025.

[45] Wani C., McCann L., Lennon M., Radu C. (2024). Digital mental health interventions for adolescents in low- and middle-income countries: Scoping review. J. Med. Internet Res..

[46] Piers R., Williams J.M., Sharpe H. (2023). Can digital mental health interventions bridge the “digital divide” for socioeconomically and digitally marginalised youth? A systematic review. Child Adolesc. Ment. Health.

[47] Mohr D.C., Silverman A.L., Youn S.J., Areán P., Bertagnolli A., Carl J., Carlton T., Chaudhary N., Cooper D., DeVito S. (2025). Digital mental health treatment implementation playbook: Successful practices from implementation experiences in American healthcare organizations. Front. Digit. Health.

[48] Hoque M., Qadri S.M., Qadri A.A., Khan M.U.H., Adedia D., Uzzaman A., Nyande F.K., Lowane M., Oladimeji O., Mokgatle M. (2026). Nexus Between Social Media Use and Mental Health Outcomes among High School Students in Kashmir, India. Ianna J. Interdiscip. Stud..

[49] World Health Organization Regional Office for Europe (2025). Addressing the Digital Determinants of Youth Mental Health and Well-Being: Policy Brief. https://www.who.int/europe/publications/i/item/WHO-EURO-2025-12187-51959-79685.

